# Uncovering the Mechanism of Quercetin in the Treatment of Premature Ovarian Failure: A Multi‐Faceted Approach Integrating Network Pharmacology, Bioinformatics Analysis and Experimental Validation

**DOI:** 10.1002/fsn3.71037

**Published:** 2025-10-09

**Authors:** Mai Zhefen, Deng Liqiang, Liu Ying, Chan Ka Kei, Li Xin, Han Xia

**Affiliations:** ^1^ Department of Gynecology Shenzhen Hospital (Futian) of Guangzhou University of Chinese Medicine Shenzhen China; ^2^ Guangzhou University of Chinese Medicine Guangzhou China; ^3^ Department of Endocrinology Shenzhen Hospital (Futian) of Guangzhou University of Chinese Medicine Shenzhen China

**Keywords:** apoptosis, KGN, oxidative stress, PI3K/AKT/P53, quercetin

## Abstract

Quercetin, a well‐recognized antioxidant, has shown potential benefits for treating premature ovarian failure (POF). This study explores the effects of quercetin on POF using network pharmacology, differential expression genes (DEGs) analysis, and in vitro experiments. Firstly, we identified quercetin targets from the TCMSP database and retrieved POF‐related targets from GeneCards, OMIM, PharmGKB, and Drugbank databases. We then identified common targets and conducted Gene Ontology (GO) and Kyoto Encyclopedia of Genes and Genomes (KEGG) enrichment analyses. Additionally, we explored the DEGs of GSE193136 and its potential signaling pathways. In vitro experiments were conducted to examine the effect of quercetin pretreatment on cell proliferation and the PI3K/AKT signaling pathway in an H_2_O_2_‐induced model. Network pharmacological analysis revealed that quercetin shares 113 common targets with POF, and GO and KEGG analyses suggested that the anti‐POF effects of quercetin may be associated with the PI3K/AKT signaling pathway and oxidative stress response. The enrichment analysis of DEGs in the GSE193136 dataset further corroborated these findings. In vitro experiments demonstrated that quercetin significantly alleviated H_2_O_2_‐induced apoptosis in KGN cells by inhibiting the expression of TP53, Caspase3, and Caspase9, followed by the enhancement of the phosphorylated expression of PI3K and AKT. This study indicates that quercetin inhibits KGN cell apoptosis by activating the PI3K/AKT signaling pathway, showing potential therapeutic benefits for POF, and may provide a theoretical basis for the application of quercetin in POF therapy.

## Graphical abstract

This study combined network pharmacology and GEO database analysis, and further confirmed the findings through in vitro experiments, suggesting that quercetin might inhibit KGN cell apoptosis by activating the PI3K/AKT signaling pathway.

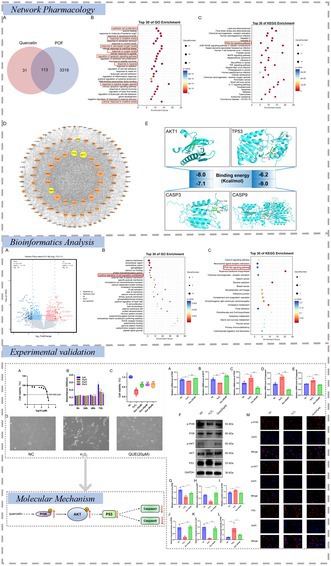

**Abbreviations**
ART: Assisted Reproductive Technology
BC: Betweenness Centrality
CC: Closeness Centrality
DC: Degree Centrality
DEGs: Differential Expression Genes
DOR: Diminished Ovarian Reserve
EC: Eigenvector Centrality
GCs: Granulosa cells
GO: Gene Ontology
HT: Hormone Therapy
KEGG: Kyoto Encyclopedia of Genes and Genomes
KGN: Human ovarian granulosa cells
LAC: Local Average Connectivity‐based Method
NOR: Normal Ovarian Reserve
POF: Premature Ovarian Failure

## 1 Introduction

Premature ovarian failure (POF) is characterized by diminished ovarian function in women under 40, with manifestations such as menstrual irregularities, elevated gonadotropin levels (FSH > 40 U/L), and reduced estrogen levels (Wu et al. 2025; Wang, Jiang, et al. 2023). Statistically, POF affects at least 1% of women in reproductive age, leading to infertility and a range of perimenopausal symptoms, including hot flushes, excessive sweating, vaginal dryness, and decreased libido (Kalantaridou et al. 1998; Penzias et al. 2020). POF can also induce psychological stress and further lower the quality of life (Silvén et al. 2022). Current treatment of POF mainly includes hormone therapy (HT) and assisted reproductive technology (ART). However, long‐term HT is associated with increased risks of complications such as blood clots, endometrial cancer, and breast cancer (Armeni et al. 2021; Rozenberg et al. 2021; Xiang et al. 2024). Additionally, the efficacy of ART is correlated to factors such as age, ovarian reserve, and overall health, and often causes physical discomfort and financial burden on patients (Zhu et al. 2023; Carson and Kallen 2021). As such, the development of accessible, affordable, and effective treatments for POF is critically important.

Quercetin, a flavonoid compound which is renowned for its antioxidant, anti‐inflammatory, antibacterial, and antiviral properties, is commonly found in various medicinal and culinary plants, including geraniums, onions, apples, and tomatoes (di Petrillo et al. 2022). Its therapeutic potential has been highlighted in numerous studies, particularly in protecting pancreatic β‐cells and mouse renal peduncle cells from apoptosis (Liu et al. 2022). A recent research study conducted by Wan et al. (Wan et al. 2024) demonstrated that quercetin significantly reduces apoptosis in human ovarian granulosa cells (KGN) via the SIRT1/SIRT3‐FOXO3 signaling pathway, suggesting its multifaceted role in treating POF (Ma et al. 2022).

The anti‐inflammatory and antioxidant properties of quercetin have been further validated by recent clinical trials, indicating its potential as a therapeutic agent for various diseases. For instance, it was demonstrated that quercetin supplementation effectively reduces the release of inflammatory cytokines, such as IL‐1α and IL‐6, in patients with diabetic nephropathy (Hickson et al. 2019). Additionally, quercetin contributes to improved insulin sensitivity in patients with polycystic ovary syndrome (PCOS), a condition often characterized by insulin resistance and metabolic disturbances, suggesting its potential role in facilitating ovarian function and overall reproductive well‐being (Rezvan et al. 2017). These findings collectively emphasize the significant anti‐inflammatory and antioxidant effects of quercetin, which are crucial for mitigating oxidative stress—a condition that can cause cellular damage and apoptosis, a key pathological mechanism in the development of POF and other reproductive disorders (Shkolnik et al. 2011; Chandimali et al. 2025; Zhang et al. 2025). The loss of granulosa cells (GCs), which are vital for ovarian function and hormone production, is primarily driven by apoptotic processes, thereby contributing to the decline in ovarian reserve and fertility (Almeida et al. 2018; Regan et al. 2018; Fan et al. 2023). However, the precise mechanism of quercetin in alleviating apoptosis in KGN cells is not being fully elucidated.

Network pharmacology is a well‐established research tool which helps to identify multiple drug targets and elucidate the corresponding pharmacological mechanisms by leveraging high‐throughput data, computational docking, and inference methods based on machine learning. Therefore, it offers mechanistic insights into the polypharmacological effects of natural compounds against diseases (Zhao et al. 2023). Additionally, the Gene Expression Omnibus (GEO) repository contains thousands of transcriptomic datasets which are generated by independent world‐class laboratories under standardized protocols (Barrett et al. 2013). By comparing differentially expressed genes (DEGs) in GCs of diminished ovarian reserve (DOR) to those with normal ovarian reserve (NOR), potential therapeutic targets for POF can be uncovered. This study integrates network pharmacology and DEGs analysis in the GEO database to identify potential pathways and targets of quercetin against POF. Besides, we will conduct in vitro experiments to verify our findings. Given its potential inhibitory effects in the apoptosis of GCs, quercetin may help to preserve ovarian function and improve reproductive outcomes for women suffering from POF.

## 2 Materials and Methods

### 2.1 Network Pharmacological Analysis

#### 2.1.1 Prediction of Related Targets of Quercetin

Potential targets of quercetin were identified using the TCMSP platform (https://tcmsp‐e.com/tcmsp.php). The corresponding target names were subsequently consolidated and standardized through UniProt (https://www.uniprot.org), ensuring the removal of redundant entries.

#### 2.1.2 Prediction of Related Targets of POF

Target genes associated with POF were queried across several databases, including GeneCards (https://www.genecards.org), OMIM (https://omim.org), PharmGKB (https://www.pharmgkb.org), and DrugBank (https://go.drugbank.com) using the keyword ‘Premature ovarian failure’. Following this, R language software was utilized to conduct an intersection analysis of the identified targets, eliminating any redundant entries.

#### 2.1.3 Exploration the Anti‐POF Target of Quercetin and Construction of the Protein–Protein Interaction (PPI) Network

The potential anti‐POF targets of quercetin were derived from the intersection of targets identified in Sections 2.1.1 and 2.1.2. The PPI network was constructed using the STRING database, specifying ‘ *Homo sapiens* ’ as the species parameter and setting a confidence score threshold of 0.4. The PPI network was then imported to Cytoscape 3.9.1 for topological analysis. With the CytoNca plugin, gene targets were under a comprehensive evaluation in which betweenness centrality (BC), degree centrality (DC), closeness centrality (CC), eigenvector centrality (EC), and local average connectivity‐based method (LAC) were collected. Core genes were identified by applying a criterion where the median of each parameter was greater than or equal to the specified threshold.

#### 2.1.4 Gene Ontology (GO) Function and Kyoto Encyclopedia of Genes and Genomes (KEGG) Pathway Enrichment Analysis

The targets identified in Section 2.1.3 were subsequently imported into R software for GO enrichment analysis and KEGG pathway analysis.

#### 2.1.5 Molecular Docking

The 2D structure of quercetin was retrieved from the PubChem database (https://pubchem.ncbi.nlm.nih.gov), and its corresponding 3D structure was generated and optimized through energy minimization using ChemBio 3D software. The 3D structures of the relevant receptor proteins were obtained by identifying key proteins in the UniProt database and acquiring their coordinates in PDB format from the RCSB PDB database (https://www.rcsb.org). Subsequently, solvent and organic molecules were removed using PyMOL software, and the structures were hydrogenated with AutoDock Tools (ADT). Both quercetin and the receptor proteins were converted into PDBQT format and docked into the active sites with parameters specifically tailored to the proteins, including size and grid center coordinates. AutoDock Vina was then employed to perform the docking of the ligands with the receptor proteins, and the resulting interactions were visualized and analyzed using PyMOL, along with the labeled amino acids.

### 2.2 DEGs Analysis of GEO Dataset

The Series GSE193136 dataset, which includes profiles of lncRNA and mRNA in GCs from patients with DOR and NOR of reproductive age, was obtained from NCBI (https://www.ncbi.nlm.nih.gov). The GEO2R online tool was utilized to identify significant DEGs by applying a stringent threshold of *p* < 0.01 and |log_2_FC| > 1.5. Subsequently, volcano plot, GO, and KEGG enrichment analyses were conducted to explore the functional implications of the DEGs.

### 2.3 In Vitro Experiment

#### 2.3.1 Materials and Reagents

The following materials and reagents were utilized in the in vitro experiments: Hydrogen peroxide solution (Sigma‐Aldrich, 88597), quercetin (MCE, 117‐39‐5), DMEM (Gibco, C11995500BT), Fetal Bovine Serum (ExCell, FSP500), PBS (Gibco, C10010500BT), Penicillin–streptomycin (Gibco, 15140122), 0.25% Trypsin (Gibco, 15050075), TRIzol (Thermo Fisher, 15596018CN), RIPA lysis buffer (Beyotime, P0013C), PhosSTOP (Roche, 4906845001), cOmplete Protease Inhibitor Cocktail (Roche, 4693124001), Hifair AdvanceFast 1st Strand cDNA Synthesis SuperMix for qPCR (DNA digester plus) (Yeasen, 11155ES60), Hieff UNICON Universal Blue qPCR SYBR Master Mix (Yeasen, 11184ES08), Pierce BCA Protein Assay Kit (Thermo Fisher, 23227), CCK‐8 Kit (Abbkine, BMU106‐CN), Immobilon Western Chemilum HRP Substrate (Millipore, WBKLS0500). Antibodies: GAPDH (CST, 2118S), p‐PI3K (Affinity, AF3241), PI3K (Affinity, AF6241), p‐AKT (ZENBIO, 381555), AKT1 (Servicebio, GB13427), P53 (Beyotime, AF7671); Goat Anti‐Rabbit (FUDE Biology, FDR007), Cy3‐labeled Goat Anti‐Rabbit IgG (Servicebio, GB21303), DAPI staining reagent (Servicebio, G1012).

#### 2.3.2 Cell Culture and Drug Treatment

KGN cells were obtained from Servicebio Co. LTD and cultured in high‐glucose DMEM medium, supplemented with 10% fetal bovine serum and 1% penicillin–streptomycin. The cells were maintained in a temperature‐controlled incubator at 37°C with 5% CO_2_. Passage and subsequent experiments were initiated when the cells reached approximately 80%–90% confluence.

For the apoptosis model of oxidative stress, KGN cells (1 × 10^4 cells/100 μL) were seeded into 96‐well plates and exposed to various concentrations of H_2_O_2_ (0, 5, 10, 50, 100, 200, 400, 600, 800, and 1000 μM) for 3 h following a 24‐h incubation. Additionally, KGN cells (1 × 10^4 cells/100 μL) were also plated onto 96‐well plates and incubated with quercetin at concentrations of 0, 20, 40, and 80 μM for durations of 0, 24, 48, and 72 h. After incubation, the medium was aspirated, and DMEM containing 10% CCK‐8 was added to each well for a 1‐h incubation. The absorbance was measured at 450 nm using a multifunctional microplate reader, and the IC_50_ value was calculated to determine the optimal H_2_O_2_ concentration for subsequent experiments and to select appropriate quercetin intervention concentrations.

Upon reaching the logarithmic growth phase, KGN cells were seeded into 96‐well plates (1 × 10^4 cells/100 μL) and divided into groups: the normal control group (NC group), the H_2_O_2_ model group (H_2_O_2_ group), and quercetin treatment groups with low, medium, and high doses (QUE 20, 40, and 80 μM). The quercetin‐treated groups were pre‐incubated with quercetin for 24 h and then exposed to 700 μM H_2_O_2_ for 3 h, while the H_2_O_2_ group was exposed to H_2_O_2_ simultaneously. Following the exposure to H_2_O_2_, the medium was replaced, and cell viability was assessed using the CCK‐8 assay.

#### 2.3.3 Real‐Time Quantitative Polymerase Chain Reaction (RT‐qPCR)

KGN cells (1 × 10^4 cells/2 mL) were seeded into 6‐well plates and divided into three groups: NC group, H_2_O_2_ group, and QUE group (20 μM QUE). Following exposure to H_2_O_2_, total RNA was extracted using TRIzol reagent, and complementary DNA (cDNA) was synthesized using Yeasen reagent (11155ES60). RT‐qPCR was performed with Yeasen reagent (11184ES08), utilizing GAPDH as the endogenous reference gene. Data analysis was conducted using the 2‐△△CT method. The sequences of the primers used are presented in Table 1.

**TABLE 1:** RT‐PCR primer sequence.

Genes	Forward (5′‐3′)	Reverse (5′‐3′)	Origin
PI3K	ACCACTACCGGAATGAATCTCT	GGGATGTGCGGGTATATTCTTC	Human
AKT1	AGCGACGTGGCTATTGTGAAG	GCCATCATTCTTGAGGAGGAAGT	Human
TP53	CAGCACATGACGGAGGTTGT	TCATCCAAATACTCCACACGC	Human
Caspase3	CAGCACATGACGGAGGTTGT	TCATCCAAATACTCCACACGC	Human
Caspase9	CTTCGTTTCTGCGAACTAACAGG	GCACCACTGGGGTAAGGTTT	Human
GAPDH	GGAGCGAGATCCCTCCAAAAT	GGCTGTTGTCATACTTCTCATGG	Human

#### 2.3.4 Immunofluorescence Staining

KGN cells were seeded, grouped, and treated as described in Section 2.3.3. Following the termination of H_2_O_2_ exposure, the cells were fixed in 4% paraformaldehyde for 15 min and permeabilized with 0.3% Triton X–100 for 10 min. Subsequently, they were blocked with 3% bovine serum albumin (BSA) for 1 h at room temperature and then incubated with the respective primary antibodies overnight at 4°C. Afterwards, the cells were treated with fluorescent secondary antibodies for 1 h at room temperature. DAPI was used for nuclear counterstaining for 10 min. The cells were then mounted with neutral gum and examined under a fluorescence microscope. At the conclusion of each procedural step, the cells were washed three times with 1× PBS to remove any residual reagents.

#### 2.3.5 Western Blotting

Following the methodology outlined in Section 2.3.4, total protein extraction from each group was conducted using RIPA lysis buffer after H_2_O_2_ exposure. Protein concentrations were quantified using a BCA protein assay kit. The isolated proteins were resolved by 10% SDS‐PAGE electrophoresis and subsequently transferred onto a PVDF membrane. The membrane was blocked with a 5% skim milk powder solution or BSA solution at room temperature for 1 h to prevent non‐specific binding. The membrane was then incubated with the appropriate antibodies (p‐PI3K, PI3K, p‐AKT, AKT, P53, GAPDH) at 4°C overnight. Following this, the membrane was washed with 1× TBST solution for three cycles of 10 min each. An HRP‐conjugated secondary antibody was then applied at room temperature for an additional hour. After washing with 1× TBST solution for another three cycles of 10 min each, the membrane was developed with an HRP chemiluminescent substrate for 1 min. Finally, the immunoblots were visualized using an automated chemiluminescence imaging system.

### 2.4 Statistical Analysis

Statistical analyses were performed using GraphPad Prism 10.1 software. Data that followed a normal distribution are presented as the mean ± standard deviation (x ± s). For comparisons among multiple groups, a one‐way analysis of variance (ANOVA) was utilized, followed by Dunnett's test. *p* < 0.05 was considered statistically significant.

## 3 Results

### 3.1 Network Pharmacology Study on the Targets and Mechanisms of Quercetin Against POF

**FIGURE 1:**
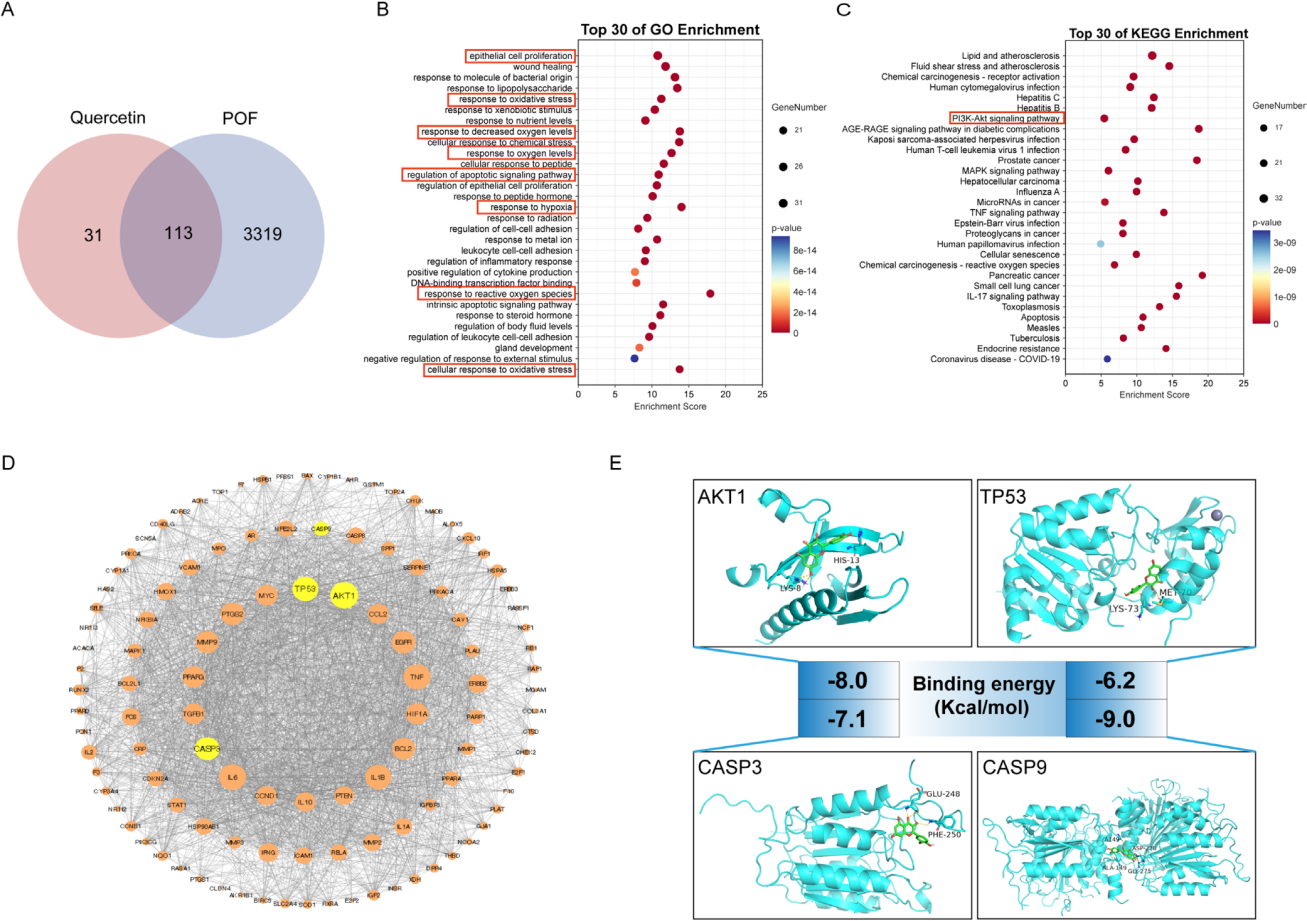
Network analysis of quercetin and POF. (A) Venn diagram of intersecting targets of quercetin and POF disease. (B) The top 30 pathways GO enrichment of core targets. (C) The top 30 pathways KEGG pathway analysis of core targets. (D) Protein–protein interactions of core targets for quercetin against POF disease. Orange circle represents the common targets between quercetin and POF, yellow circle represents the core targets, and the size of the circle represents the level of degree. (E) Molecular docking of AKT1, TP53, CASP3, and CASP9 with quercetin respectively.

A total of 144 quercetin targets were retrieved from the TCMSP database, while 3432 POF‐related targets were collected from the GeneCards, OMIM, PharmGKB, and DrugBank databases (Data S1 and S2). The intersection of quercetin and POF‐related targets was illustrated using a Venn diagram, identifying 113 common targets (Figure 1A). The intersecting targets were analyzed by R software for enrichment analysis. A total of 2295 GO functional enrichment items were identified (*p* < 0.05), with the top 30 items ranked by Gene Ratio presented in Figure 1B, in which significant involvement with oxidative stress responses was observed. Furthermore, KEGG pathway analysis revealed that the 113 targets were associated with 172 signaling pathways. The top 30 pathways, ordered by Gene Ratio, are presented in Figure 1C, emphasizing critical roles in pathways related to the PI3K‐Akt signaling pathway, the AGE‐RAGE signaling pathway in diabetic complications, and other notable pathways.

The 113 targets were subsequently imported into the STRING database for PPI network analysis, resulting in the PPI network diagram. The core genes identified included AKT1, TP53, CASP3, and CASP9, etc. (Figure 1D). Molecular docking analyses were performed to assess the affinity between quercetin and the proteins encoded by AKT1, TP53, CASP3, and CASP9. As shown in Figure 1E, the binding energies of quercetin with AKT1, TP53, CASP3, and CASP9 were −8.0, −6.2, −7.1, and −9.0 kcal/mol, respectively. These findings suggest that quercetin exhibits a favorable binding affinity for these proteins.

### 3.2 DEGs Analysis of the GSE193136 Dataset

**FIGURE 2:**
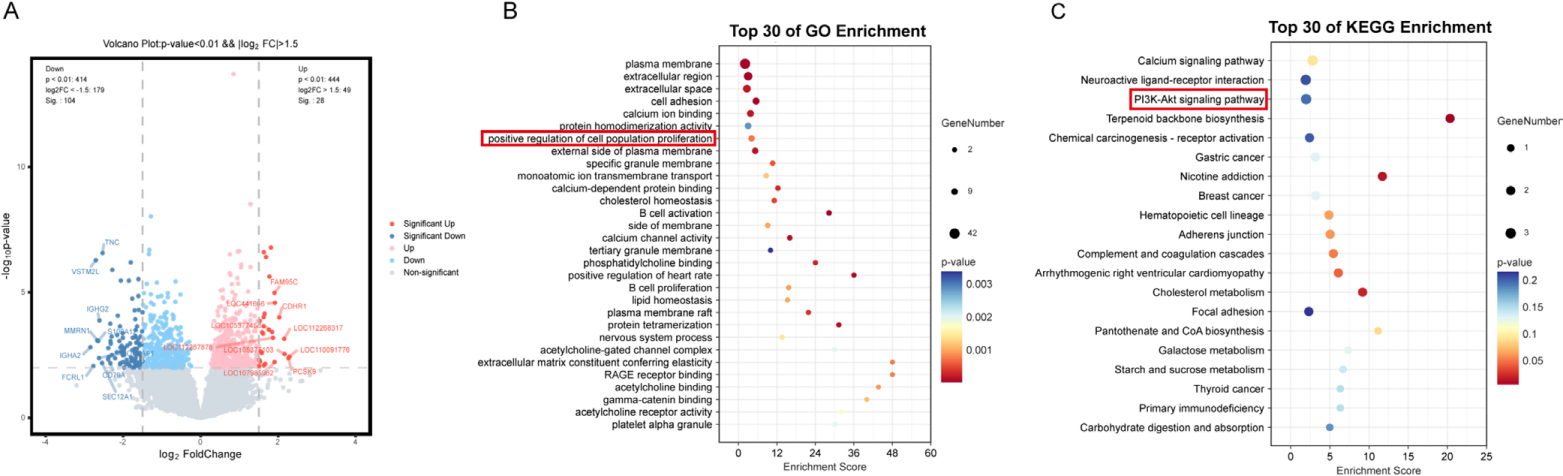
Differential gene analysis of GEO database for DOR. (A) Volcano plot of 132 DEGs of GCs in GEO dataset between DOR and NOR patients of reproductive age, 104 for downregulated and 28 for up‐regulated. *p* < 0.01, |log_2_ FC| > 1.5. (B) The top 30 pathwaysGO enrichment analysis of the selected DEGs. (C) The top 30 pathways KEGG pathway analysis of the selected DEGs.

The GSE193136 dataset was analyzed using the criteria of *p* < 0.01 and |log_2_FC| > 1.5, resulting in the identification of 132 DEGs, comprising 104 downregulated and 28 up‐regulated genes in comparison to the NOR group (Figure 2A). GO functional analysis showed that the DEGs were predominantly associated with the biological process of cell proliferation regulation (Figure 2B). Additionally, KEGG pathway analysis demonstrated significant enrichment of the DEGs in the Calcium signaling pathway, Neuroactive ligand‐receptor interaction, and the PI3K‐AKT signaling pathway (Figure 2C). These results further support the enrichment analysis of network pharmacology.

### 3.3 Quercetin Alleviates H_2_O_2_ ‐Induced KGN Cell Injury and Morphological Changes

**FIGURE 3:**
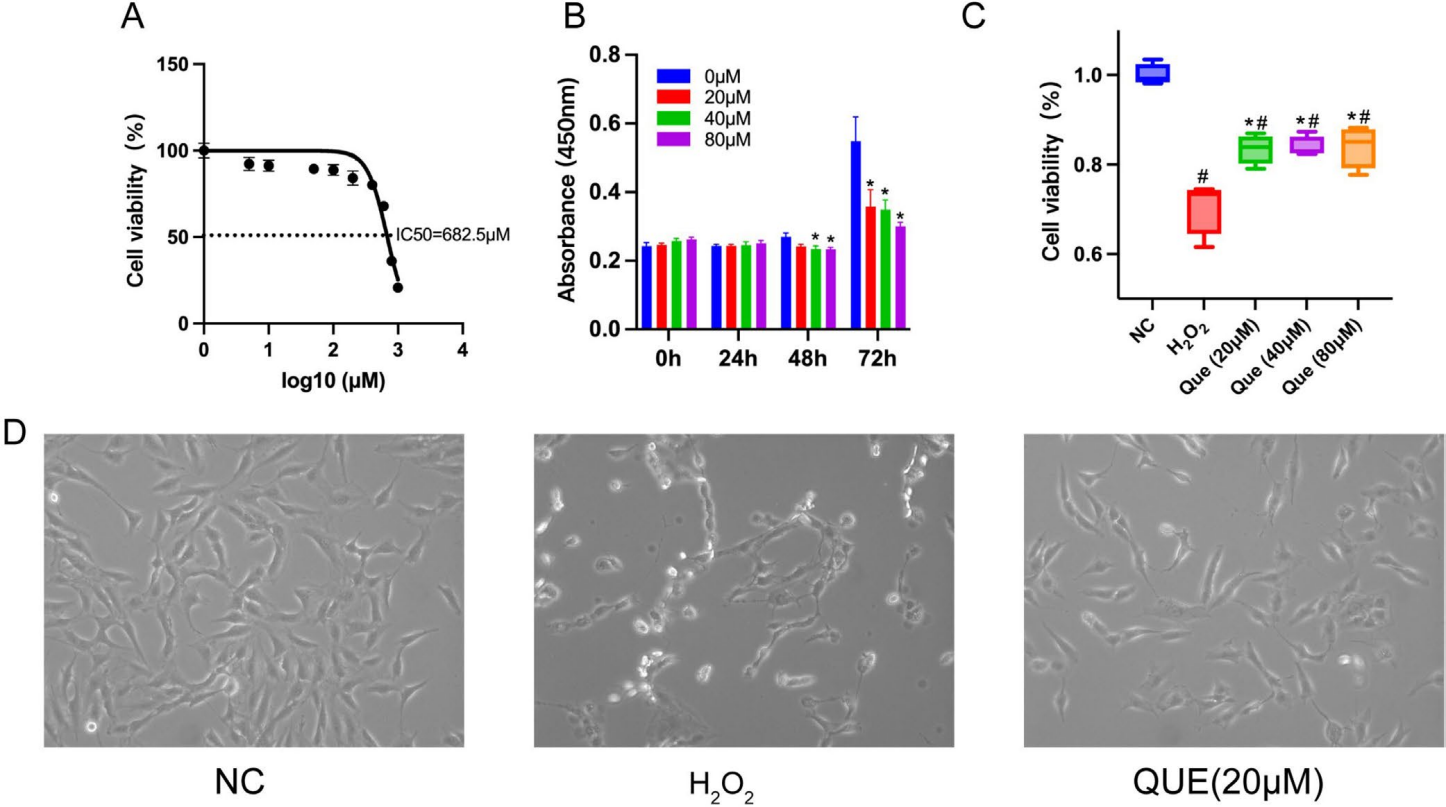
Quercetin alleviated H_2_O_2_ induced KGN cell injury, altered cell morphology, and mRNA level expression. (A) The cytotoxicity of H_2_O_2_ (0–1000 μM) treatment for 3 h on cultured KGN cells by CCK‐8 assay, *n* = 4 per group. (B) The cytotoxicity of quercetin (0–80 μM) treatment for 0 h, 24 h, 48 h, 72 h on cultured KGN cells by CCK‐8 assay, *n* = 4 per group. *p < 0.05 versus 0 μM group. (C) Protective effect of quercetin (20, 40, 80 μM) against H_2_O_2_‐induced KGN cell injury, *n* = 4 per group. #*p* < 0.05 versus NC group, *p < 0.05 versus H_2_O_2_ group. (D) Effect of quercetin on H_2_O_2_‐induced KGN cell morphology, observed under optical microscopy (100X).

By integrating the results of network pharmacology, we established an apoptosis model of oxidative stress using H_2_O_2_ and treated the cells with quercetin to assess its effects on cell proliferation and modulation of the PI3K‐AKT signaling pathway in KGN cells. The half‐maximal inhibitory concentration (IC_50_) of KGN cells exposed to varying concentrations of H_2_O_2_ for 3 h was determined to be 682.5 μM (Figure 3A). To facilitate subsequent experimental calculations, a concentration of 700 μM H_2_O_2_ was selected for the induction of the H_2_O_2_ model group. The viability of KGN cells remained stable after 24 h of treatment with various concentrations of quercetin. However, a significant decrease in cell viability was observed in the 40 and 80 μM quercetin groups at 48 h. As depicted in Figure 3B, cell viability in the 20, 40, and 80 μM quercetin groups was significantly reduced at 72 h. In addition, following a 24‐h pre‐treatment with 20, 40, and 80 μM quercetin, the cell viability in all quercetin‐treated groups was significantly higher compared to the H_2_O_2_ group after H_2_O_2_‐induced injury (Figure 3C). Furthermore, the 20 μM QUE group significantly decreased KGN apoptosis (Figure 3D). To minimize quercetin‐induced cytotoxicity while achieving measurable effects, we selected 24 h as the optimal treatment duration and 20 μM quercetin for subsequent experiments.

### 3.4 Quercetin Regulates Gene Expression and Protein Expression Associated With Apoptosis and the PI3K/AKT/P53 Pathway in H_2_O_2_ ‐Induced KGN Cells

**FIGURE 4:**
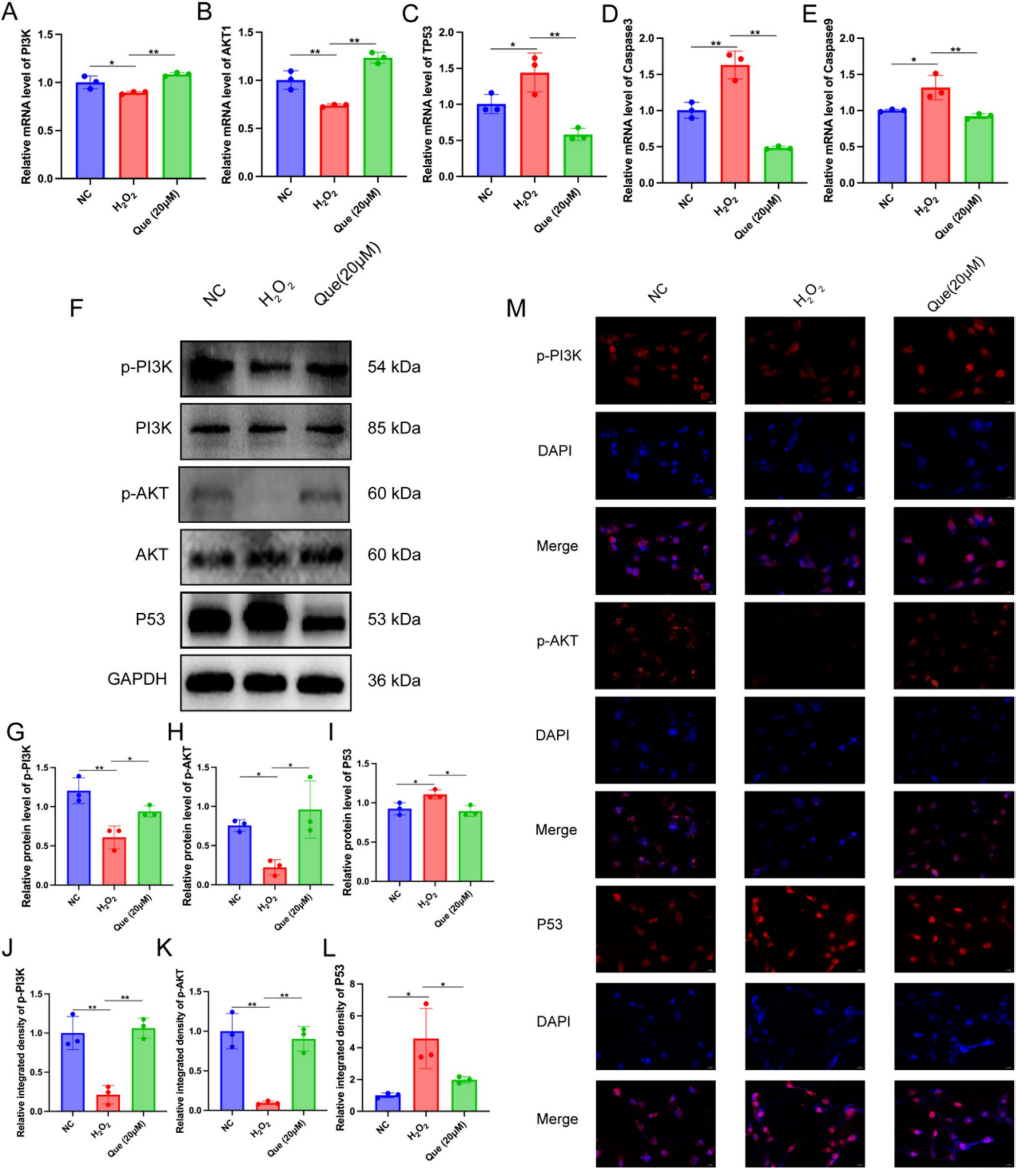
Quercetin regulated apoptosis‐related mRNA and protein expression of H_2_O_2_‐induced KGN cell. (A–E) The mRNA expression of PI3K, AKT1, TP53, Caspase3, and Caspase9 in quercetin treatment KGN cells with H_2_O_2_‐induced, *n* = 3 per group. *p < 0.05, **p < 0.01 versus H_2_O_2_ group. (F–I) Protein levels of p‐PI3K, PI3K, p‐AKT, AKT and P53 under western blot analysis, *n* = 3. (J–M) Representative images and relative integrated density of p‐PI3K, p‐AKT and P53 immunofluorescence staining. Scale bar = 20 m, *n* = 3. *p < 0.05, **p < 0.01, versus the H_2_O_2_ group.

We found that quercetin may intervene in POF by regulating the PI3K/AKT/P53 signaling pathway through network pharmacology and bioinformatics analysis. Based on the changes in cell proliferation, morphology, and phenotype, we further verified its potential mechanism. We found that quercetin pretreatment significantly improved the gene expression of PI3K and AKT1 (Figure 4A,B). In addition, the mRNA levels of TP53, Caspase3, and Caspase9 were considerably increased after H_2_O_2_ stimulation for 3 h, whereas expressions were reduced by quercetin pretreatment (Figure 4C–E). Additionally, the levels of phosphorylated PI3K (p‐PI3K) and phosphorylated Akt (p‐Akt) were markedly reduced in the H_2_O_2_ group compared to the NC group, while the level of P53 protein was significantly elevated. Conversely, the protein expression levels of p‐PI3K and p‐Akt were significantly upregulated, and the P53 protein level was notably downregulated when the KGN cells were pretreated with quercetin before the stimulation of H_2_O_2_ (Figure 4F–I). Furthermore, we performed immunofluorescence staining, which also confirmed that quercetin could activate the phosphorylation of p‐PI3K and p‐AKT, inhibit the protein levels of P53 (Figure 4J–M). In summary, we found that quercetin could inhibit the expression of P53 by activating the phosphorylation of the PI3K/AKT signaling pathway, thus inhibiting the apoptosis of GCs.

## 4 Discussion

Quercetin is increasingly recognized as a potent antioxidant with significant potential for mitigating oxidative stress‐related damage, particularly in the context of POF. Early studies have shown that quercetin markedly elevates sex hormone levels in rat models of POF, promotes follicular development, and stimulates the proliferation of GCs, thereby improving ovarian function (Zheng et al. 2022; Vollmannová et al. 2024). These findings highlight the role of quercetin in enhancing reproductive health and its therapeutic potential in addressing POF. The apoptosis of follicular membrane cells is critical for ovarian health and functionality. Excessive apoptosis can lead to follicular atresia, delayed development of dominant follicles, and reduced secretion of estrogen and anti‐Müllerian hormone, making it essential to understand how quercetin inhibits GCs' apoptosis, thus preserving ovarian function (Hou et al. 2021; Qin et al. 2022; Zhang et al. 2024).

By utilizing network pharmacology and bioinformatics analysis, we identified targets and pathways associated with quercetin in the context of POF and evaluated its binding affinity to proteins through molecular docking (Li and Zhang 2013; Nogales et al. 2022). Our analysis revealed 113 common targets between quercetin and POF, with AKT1, TP53, Caspase3, and Caspase9 emerging as key genes. The PI3K/AKT signaling pathway was identified as a primary pathway in our KEGG enrichment analysis, which is further supported by the bioinformatics analysis of DEGs. AKT, as a downstream target of the PI3K/AKT pathway, plays a crucial role in regulating cell proliferation, differentiation, and apoptosis (Green et al. 2013; Yu and Cui 2016). Increased phosphorylation of AKT has been shown to inhibit GCs apoptosis and enhance ovarian function (Lei et al. 2018; Lin and Dai 2022; Dai et al. 2023). The activation of AKT also suppresses the expression of TP53, a vital tumor suppressor gene that regulates cell cycle progression and apoptosis in response to cellular stress and DNA damage (Zhang et al. 2022; Chibaya et al. 2021). Once activated, P53 can initiate a caspase cascade, which further facilitates the amplification and execution of the apoptotic signal, culminating in cell apoptosis (Voskarides and Giannopoulou 2023; Lin et al. 2015; Wang et al. 2019; Wang, Yan, et al. 2023; McArthur et al. 2018). Among these, Caspase9 serves as an initiator caspase that is vital for the intrinsic pathway of apoptosis (Johnson and Jarvis 2004). In response to DNA damage or various stress signals, mitochondria release cytochrome c, which then binds to apoptotic protease activator 1 (Apaf‐1), leading to apoptosome formation. The apoptosome effectively recruits and activates pro‐caspase9, transforming it into its active variant. Subsequently, activated Caspase9 proceeds to activate and cleave pro‐caspase3, resulting in the formation of fully active Caspase3 (Shakeri et al. 2017; Jemmerson et al. 2021). This activated form of Caspase3 then cleaves a variety of substrates, such as poly‐(ADP‐ribose) polymerase (PARP), which results in the loss of DNA repair capabilities and ultimately triggers apoptosis (Ruemmele et al. 1999; Eskandari and Eaves 2022). Thus, the anti‐POF effect of quercetin may be significantly associated with the modulation of these apoptotic pathways.

In our study, we employed KGN cells, a particular type of cells which derived from an ovarian granulosa cell tumor, to investigate the regulatory mechanisms of quercetin in apoptosis. We established an in vitro model of H_2_O_2_‐induced apoptosis to simulate oxidative stress conditions. The introduction of H_2_O_2_ led to cellular apoptosis that was characterized by mitochondrial dysfunction and activation of apoptotic pathways (Liang et al. 2024; Yuan et al. 2024). Morphological analyses revealed that H_2_O_2_ treatment resulted in reduced cell size and number, while quercetin pretreatment effectively mitigated these changes. To further elucidate the apoptotic signaling modifications and their mediation through the PI3K/AKT pathway, we assessed mRNA expression levels of key markers. Our results demonstrated that H_2_O_2_ exposure significantly increased the mRNA levels of TP53, Caspase3, and Caspase9, while quercetin pretreatment counteracted these effects. Moreover, quercetin pretreatment elevated PI3K and AKT1 mRNA levels during H_2_O_2_ exposure in KGN cells. Previous studies have indicated that quercetin can enhance the phosphorylation of PI3K and AKT, thereby promoting recovery from neurological deficits in models of cerebral ischemia/reperfusion injury (Li et al. 2023). Our findings revealed a significant decrease in the expression of p‐PI3K and p‐AKT, while the levels of TP53, Caspase3, and Caspase9 were found to be increased. After quercetin pretreatment, the levels of p‐PI3K and p‐AKT in KGN cells were significantly upregulated compared to the H_2_O_2_ group, whereas the levels of TP53, Caspase3, and Caspase9 were downregulated compared to those in the H_2_O_2_ group. In other words, quercetin has been shown to activate the PI3K/AKT signaling pathway, leading to the downregulation of TP53, Caspase3, and Caspase9 expression, thus preventing apoptosis in KGN cells.

However, owing to a limited budget, the effects of quercetin were shown in vitro and its effect on apoptosis in GCs was demonstrated by immunofluorescence staining only. Future research should explore the effect of quercetin on POF in an in vivo model in addition to the use of PI3K inhibitors, so as to further validate its role in the regulation of the PI3K/AKT/P53 pathway. Furthermore, methods such as Hoechst staining, flow cytometry, or Western blot should be employed in the apoptosis experiments.

## 5 Conclusion

By integrating network pharmacology, bioinformatics analysis of DEGs, and experimental validation, it is demonstrated that quercetin exerts anti‐apoptotic effects in KGN cells by inhibiting the expression of TP53, Caspase3, and Caspase9 through the activation of the PI3K/AKT signaling pathway. Based on our findings, we suggest that the effectiveness of quercetin in patients with POF can be examined by conducting clinical trials and to provide clinical evidence as a complementary treatment for POF.

## Author Contributions

Xia Han provided the conception and design of the study and takes responsibility for the integrity of the data and the accuracy of the data analysis. Zhe‐fen Mai participated in the design and execution of the study and wrote the manuscript. Li‐qiang Deng and Ying Liu supervised the execution of the study and revised the manuscript critically for important intellectual content. Chan Ka Kei collected and analyzed data repeatedly to ensure accuracy, and Chan Ka Kei checked the grammar of the entire article. All authors have read and approved the final version of the manuscript.

## Conflicts of Interest

The authors declare no conflicts of interest.

## Supporting information


**Data S1:** Target name and according gene name of quercetin from TCMSP database.

**Data S2:** Gene target of POF from GeneCards, OMIM, PharmGKB and Drugbank database.

**Data S3:** DEGs of GSE193136 dataset (*p* < 0.01, |log_2_ FC| > 1.5).

## Data Availability

The data that support the findings of this study are available from the corresponding author upon reasonable request.
